# Effects of the neonicotinoid acetamiprid in syrup on *Bombus impatiens* (Hymenoptera: Apidae) microcolony development

**DOI:** 10.1371/journal.pone.0241111

**Published:** 2020-10-29

**Authors:** Allison A. Camp, Wanda C. Williams, Brian D. Eitzer, Robert W. Koethe, David M. Lehmann

**Affiliations:** 1 ORISE Researcher, Oak Ridge Associated Universities, Research Triangle Park, North Carolina, United States of America; 2 Public Health & Integrated Toxicology Program, Cardiopulmonary & Immunotoxicology Branch, Center for Public Health and Environmental Assessment (CPHEA), US - Environmental Protection Agency, Research Triangle Park, North Carolina, United States of America; 3 The Connecticut Agricultural Experiment Station, New Haven, Connecticut, United States of America; 4 Region 1 Office, Land, Chemicals and Redevelopment Division RCRA, Waste Management and Pesticides Section US – Environmental Protection Agency, Boston, Massachusetts, United States of America; 5 Public Health & Environmental Systems Division, Exposure Indicators Branch, Center for Public Health and Environmental Assessment (CPHEA), US - Environmental Protection Agency, Research Triangle Park, North Carolina, United States of America; Institut Sophia Agrobiotech, FRANCE

## Abstract

Worldwide, many pollinator populations are in decline. Population reductions have been documented for the agriculturally important honey bee (*Apis mellifera*), and other bee species such as bumble bees that are also critical for pollinating crops and natural landscapes. A variety of factors contribute to the observed population reductions, including exposure to agrochemicals. In recent decades, neonicotinoid pesticide use has dramatically increased, as have concerns regarding the safety of these chemicals for pollinator health. Here we assessed the toxicity of the neonicotinoid acetamiprid to the bumble bee *Bombus impatiens*, a species commercially available for use in agricultural settings in North America. Using the microcolony model, we examined nest growth, development and subsequent nest productivity as measured by drone production. We found that high concentrations of acetamiprid in syrup (11,300 μg/L) significantly impacted nest growth and development, and ultimately drone production, and exposure to 1,130 μg/L acetamiprid also significantly decreased drone production. The no observable adverse effect level was 113 μg/L. Overall, acetamiprid delivered in syrup can negatively impact *B*. *impatiens* nest development and productivity, however only at concentrations above which would be expected in the environment when used according to label rates.

## Introduction

Worldwide, bees are responsible for the majority of pollination services necessary to maintain crops and sustain agricultural systems [1, 2]. Despite their importance, many bee populations are in decline due to complex factors such as habitat loss [3, 4], pesticide use [5, 6], parasites [6, 7], and pathogens [2]. In the United States, eight bee species have been listed as endangered, and population declines have been documented around the country [8–11].

While honey bees (*Apis mellifera*) provide critical pollination services in agricultural settings, other bees, such as bumble bees (*Bombus* sp) also make important contributions. Bumble bees are used commercially to pollinate a variety of crops including lowbush blueberries [12, 13], greenhouse tomatoes [14, 15], and peppers [16]. In many cases, bumble bees are more effective pollinators than honey bees due to differences in body size, morphological features, pollination style, and foraging habits [12, 17, 18]. For instance, bumble bees can sonicate flowers to obtain pollen and can forage in more inclement conditions than honey bees [17, 19, 20].

Neonicotinoid pesticides, which target the insect nicotinic acetylcholine receptor and disrupt nervous system function, are widely used in agriculture to control sucking insects (e.g., aphids, thrips) [21–23]. There are two classes of neonicotinoids, the nitro-group containing (imidacloprid, clothianidin, thiamethoxam, dinotefuran, nitenypyram) and the cyano-group containing (acetamiprid and thiacloprid) [24]. Neonicotinoids in the nitro-group containing class are known to have adverse impacts on pollinators and have been subject to bans in the European Union [24–26]. Neonicotinoid research with bumble bees has also focused on the nitro-group containing class, and results indicate that these pesticides have adverse impacts on growth, behavior, and survival [19, 27–29]. Because the acute toxicity of cyano-neonicotinoids to bees is known to be lower than that of nitro-group neonicotinoids [30], comparatively little research effort has been dedicated to the impacts of thiacloprid and acetamiprid on pollinators.

Acetamiprid is approved for use on a wide variety of crops that bumble bees pollinate, including many types of fruit, and is applied as a foliar spray [21, 31]. As such, nectar and/or pollen may become contaminated with acetamiprid [32–34]. Acetamiprid exposure may pose a threat to wild and managed bumble bee populations, however, very little data exists for the effects of chronic acetamiprid exposure on pollinator health, and of the sparse research, most studies have focused on honey bees.

The microcolony model is a method of assessing bumble bee sensitivity to stressors that has broad utility [35]. In this experimental design, confined bumble bee workers without a true queen establish a false queen that lays unfertilized eggs (i.e., eggs that will become drones). The organized workers will build nest structures and tend to brood akin to a queenright colony, however, unlike queenright colonies, microcolonies are suitable for large scale experiments. Microcolonies can be evaluated more easily and reliably, thus functioning as a useful experimental format for assessing nest-relevant endpoints that have population-level implications [36].

Previously, we studied the effects of acetamiprid delivered in pollen on *B*. *impatiens* microcolony development and demonstrated that acetamiprid negatively impacts reproductive endpoints, but only when tested at concentrations substantially higher than expected environmental concentrations [37]. In this work, we assessed the chronic toxicity of acetamiprid delivered in syrup to microcolonies of *B*. *impatiens*. We examined microcolony growth and development over six weeks in order to observe impacts on brood development, drone production and worker survival. This work is one of the few assessments of chronic acetamiprid exposure for *B*. *impatiens* and provides critical foundational knowledge about a commonly used neonicotinoid pesticide.

## Materials and methods

### Chemicals and reagents

Acetamiprid (purity 99.9%) was obtained from Sigma Aldrich and used for chemical exposures (CASRN 135410-20-7; St. Louis, MO). Chemicals used to produce inverted syrup were sorbic acid (Amresco, Dallas, TX), citric acid anhydrous (Fisher Scientific, Hampton, NH), pure cane sugar (Domino Foods, Inc) and distilled water (Gibco, Gaithersburg, MD). Analytical chemistry methods used acetonitrile, magnesium sulfate, sodium acetate, toluene and formic acid (Fisher Scientific, Hampton, NH), as well as DSC-C18 and PSA (Supelco, Millipore Sigma, Burlington, MA).

### Bumble bee stock

Newly emerged (<24 hrs old) *B*. *impatiens* workers (Biobest^®^, Romulus, MI) were acclimated to laboratory conditions for 24 hrs prior to microcolony initiation. Workers were provisioned with 50/50 inverted sugar syrup (50% glucose and 50% fructose) prepared in distilled water and contained 2.95 mM citric acid and 8.32 mM sorbic acid. Pollen paste (2.5 g pollen/1 mL 50/50 inverted syrup), prepared from honey bee corbicular pollen (collected 2015 from ornamental nurseries, Connecticut, USA), was also provided. Bees were maintained in an environmental chamber (Percival, Perry, IA) in continuous darkness at 60 ± 1% relative humidity and 25 ± 0.2°C during acclimation and for the duration of the experiment.

### Microcolony initiation, exposures, and monitoring

Microcolony initiation procedures, test chambers, pollen and syrup delivery methods, as well as feeding schedule have been described previously [37]. Acetamiprid (1.13, 11.3, 113, 1,130, 11,300 μg/L) formulated in sugar syrup was delivered in syrup feeders upon initiation of microcolonies and renewed Monday, Wednesday, and Friday for the duration of the experiment. Control microcolonies were provided plain sugar syrup. As with previous microcolony experiments, pollen and syrup consumption were tracked throughout the experiment. The microcolonies were maintained for 6 weeks and were monitored daily for adult worker mortality, appearance of egg chambers, capped egg chambers, brood masses, pupal cells, and drone emergence. Worker behavior was also observed daily for qualitative changes in activity (e.g., lethargic or hyperactive). Eclosed drones were removed from the microcolonies upon emergence and weighed. Microcolony development of one randomly selected microcolony per treatment group was photographically documented using a digital camera (iPhone 6, Apple, Cupertino, CA). Ten microcolonies were used for each experimental group and one evaporation control microcolony (no bees present) was run in parallel to establish background evaporation for syrup and pollen paste.

### Sample preparation for analytical chemistry

Syrup samples were sent for chemical analysis to validate the nominal concentrations as well as determine degradation between syringe replacement. Samples for chemistry were taken immediately after working syrup solutions were prepared (0 hrs) and 48 hrs after preparation for day 0 and 2 of the experiment. Samples were stored at -20°C until analysis.

The extraction procedure was adapted from that used to analyze nectar from squash samples by Eitzer and Stoner [38] though due to much higher concentrations additional dilutions and external standards were used to keep it in the calibration range. Instrumental conditions were also modified as analyzed on a different LC-MS system. For analysis, 0.5–1.5 g of the syrup samples were weighed out and combined with reverse osmosis water to a final weight of 15 g. Then, 15 mL of acetonitrile, 6 g magnesium sulfate and 1.5 g sodium acetate were added to the samples. The resulting mixtures were thoroughly shaken and centrifuged. Next, 10 mL portions of the acetonitrile eluent were transferred to a centrifuge tube containing 2 mL toluene, 1.5 g magnesium sulfate, 0.5 g DSC-C18 and 0.5 g PSA, then vortexed. Tubes were centrifuged and depending on the expected sample concentration the eluents were either diluted, analyzed without dilution, or concentrated under nitrogen. The resulting solutions were filtered into glass vials for LC-MS analysis.

### LC-MS analysis

Samples were analyzed on Dionex 3000 liquid chromatograph (Dionex, Sunnyvale, CA) interfaced to a Q-Exactive mass spectrometer (Thermo Scientific, Waltham, MA) using a Agilent SB-C18 RRHD 2.1 x 150 mm, 1.8 (Agilent Technologies, Santa Clara, CA) u column held at 40°C. Mobile phase A was water with 0.1% formic acid and mobile Phase B was acetonitrile with 0.1% formic acid. A 1 μL sample was injected using a gradient starting at 95% A going to 5% A over 16 mins and then held at 5% A for 4 mins. The mass spectrometer was operated in a positive electrospray mode at 3.5 Kv. Three scans were used, a full scan from 100–1200 m/z at a resolution of 140,000 and two all ion fragmentation scans (at NCEs of 15 and 45) at a resolution of 35,000. Samples were quantified in the full scan using a 5 ppm window around the theoretical mass of the (M+H)^+^ ion. Samples were quantified using external standards and a 5-point calibration curve between 5 and 200 ng/g.

### Data processing and statistical analysis

Syrup and pollen consumption values were corrected for evaporation, and values that were negative after evaporation adjustment were assumed to be zero. If feeding syringes leaked, leading to inaccurate syrup consumption values, the inaccurate value was omitted and replaced with the average syrup consumption for that treatment group and day. Microcolonies that perished prior to the termination of the experiment (one within the 11.3 μg/L group, four within the 1,130 μg/L group) were omitted from analyses for which a complete data set was necessary. Estimates of acetamiprid consumption on a per bee basis were corrected for mortality. Differences between the control and treatment groups were analyzed with One-way Analysis of Variance (ANOVA) and Dunn’s multiple comparison test when variances were no different. When variances were significantly different according to the Brown-Forsythe or Bartlett’s tests, a non-parametric test was used (Kruskal-Wallis with Dunnett’s multiple comparisons test). Data are presented as mean ± standard deviation (SD) and statistical significance was defined as p<0.05. All statistical analyses were performed with GraphPad Prism^®^ (v6; La Jolla, CA).

## Results

### Microcolony workers

Average weights of initiating workers for each treatment were compared, and average starting worker weights for 1,130 and 11,300 μg/L microcolonies were significantly lower (0 μg/L: 181 ± 19 mg, 1.13 μg/L: 186 ± 13, mg 11.3 μg/L: 183 ± 14 mg, 113 μg/L: 170 ± 22 mg, 1130 μg/L: 158 ± 6 mg, 11300 μg/L: 154 ± 12 mg; p<0.05, Kruskal-Wallis with Dunn’s multiple comparisons test). However, at the termination of the microcolonies, there were no differences in average worker weights (0 μg/L: 198 ± 29 mg, 1.13 μg/L: 206 ± 22, mg 11.3 μg/L: 212 ± 55 mg, 113 μg/L: 197 ± 38 mg, 1,130 μg/L: 217 ± 13 mg, 11,300 μg/L: 200 ± 22 mg; p>0.05, Kruskal-Wallis with Dunn’s multiple comparisons test).

**Fig 1:**
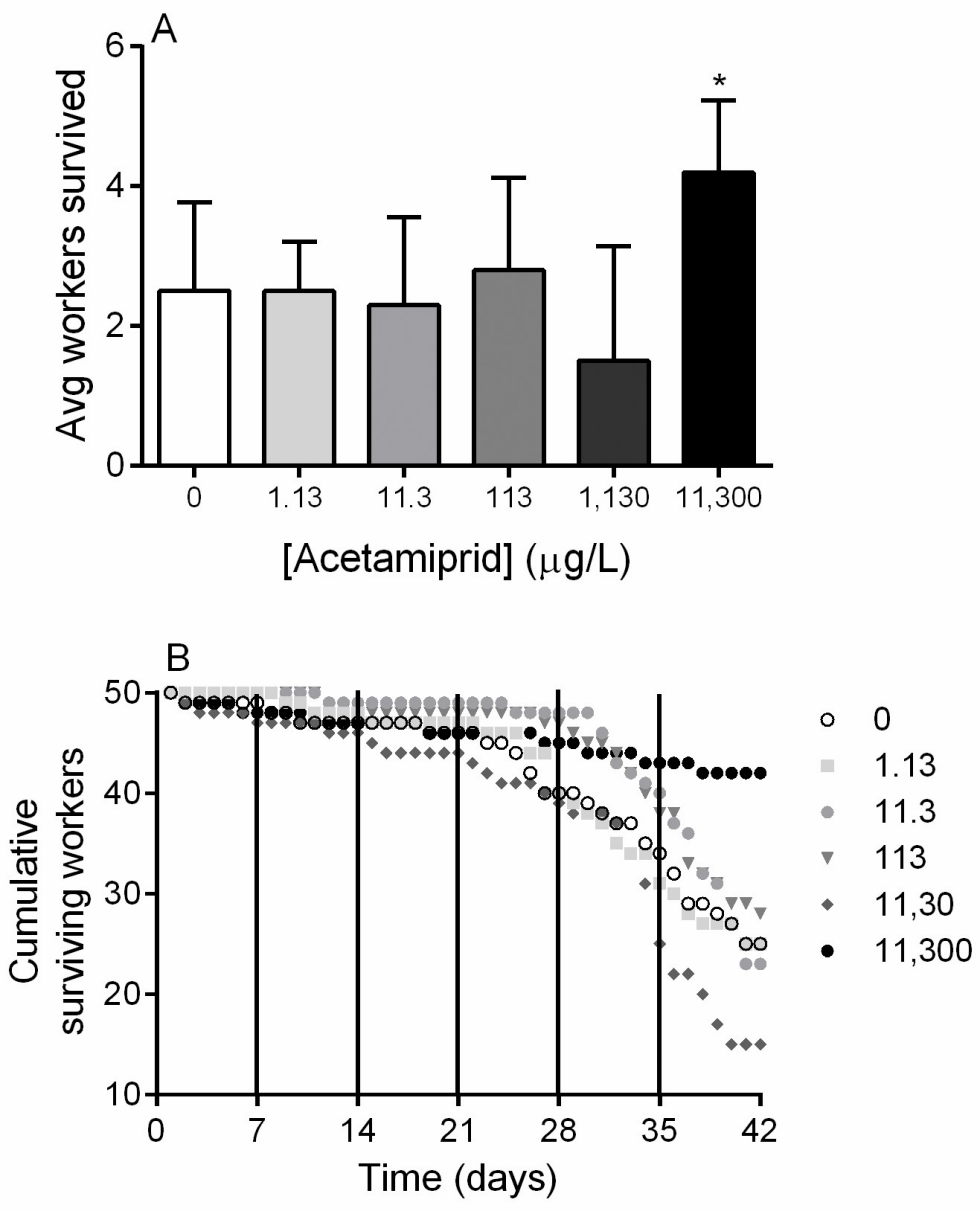
Microcolony founding worker survival. (**A**) Average worker survival at termination of the experiment. (**B**) Worker survival over time for each exposure group. Acetamiprid concentrations are expressed in μg/L. Data shown as mean ± SD (n = 10). * denotes p<0.05.

Worker survival was documented throughout the six-week experiment. Notably, the highest concentration group (11,300 μg/L) had significantly higher survival than the control group (p<0.05, One-Way ANOVA with Dunnett’s multiple comparisons test; Fig 1A). Cumulative survival for each group was examined, and week four marked the beginning of a downward survival trend for the groups that experienced notable mortality (Fig 1B).

Workers were also observed daily for altered behavior (changes in activity). Occurrences of altered behavior were observed only in the two highest exposure concentrations, and incidence of at least one bee displaying altered behavior occurred 4.8 ± 3.3% of the days for 1,130 μg/L and 24.1 ± 19% of the days for 11,300 μg/L.

### Microcolony development

**Fig 2:**
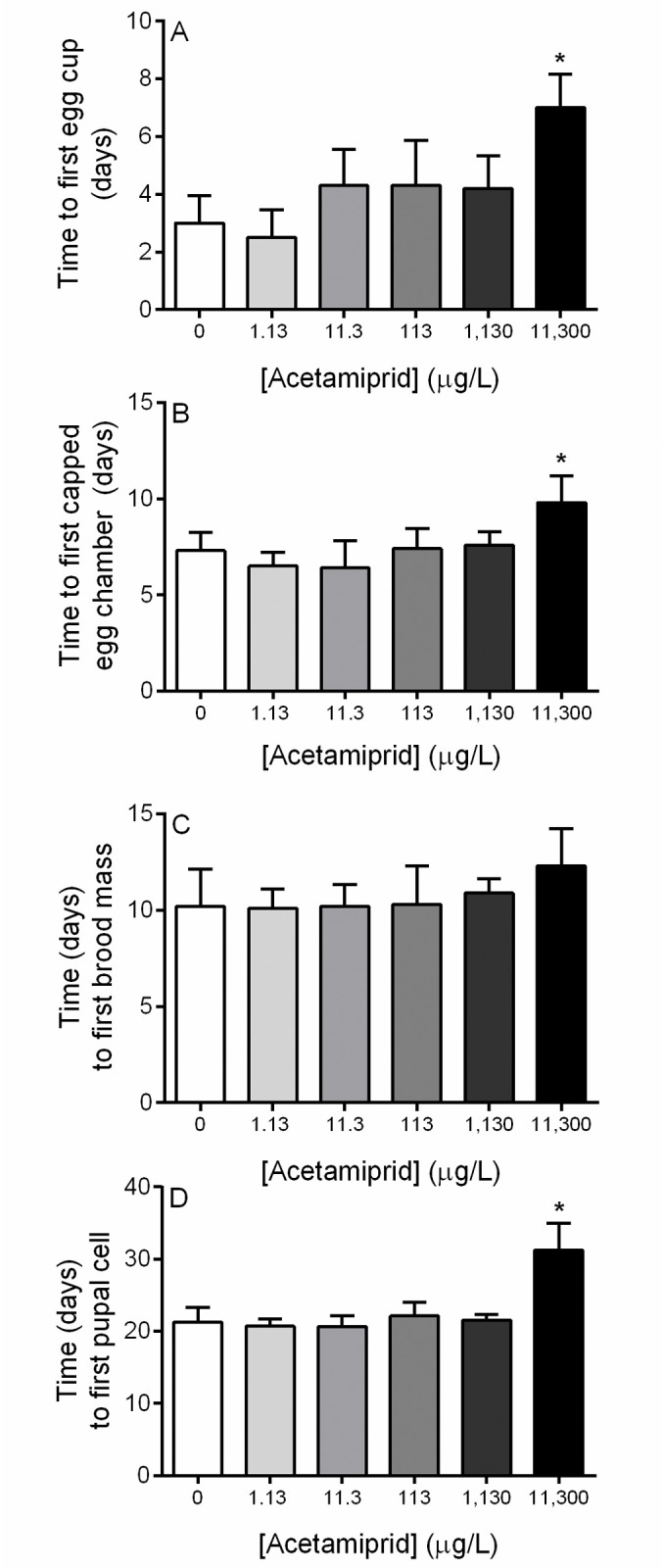
*B*. *impatiens* microcolony nest development with acetamiprid exposure. (**A**) Time to first egg cup. (**B**) Time to first capped egg chamber. (**C**) Time to first brood mass. (**D**) Time to first pupal cell. Observations were conducted daily for the duration of the experiment. Data shown as mean ± SD (n = 10). * denotes p<0.05.

**Fig 3:**
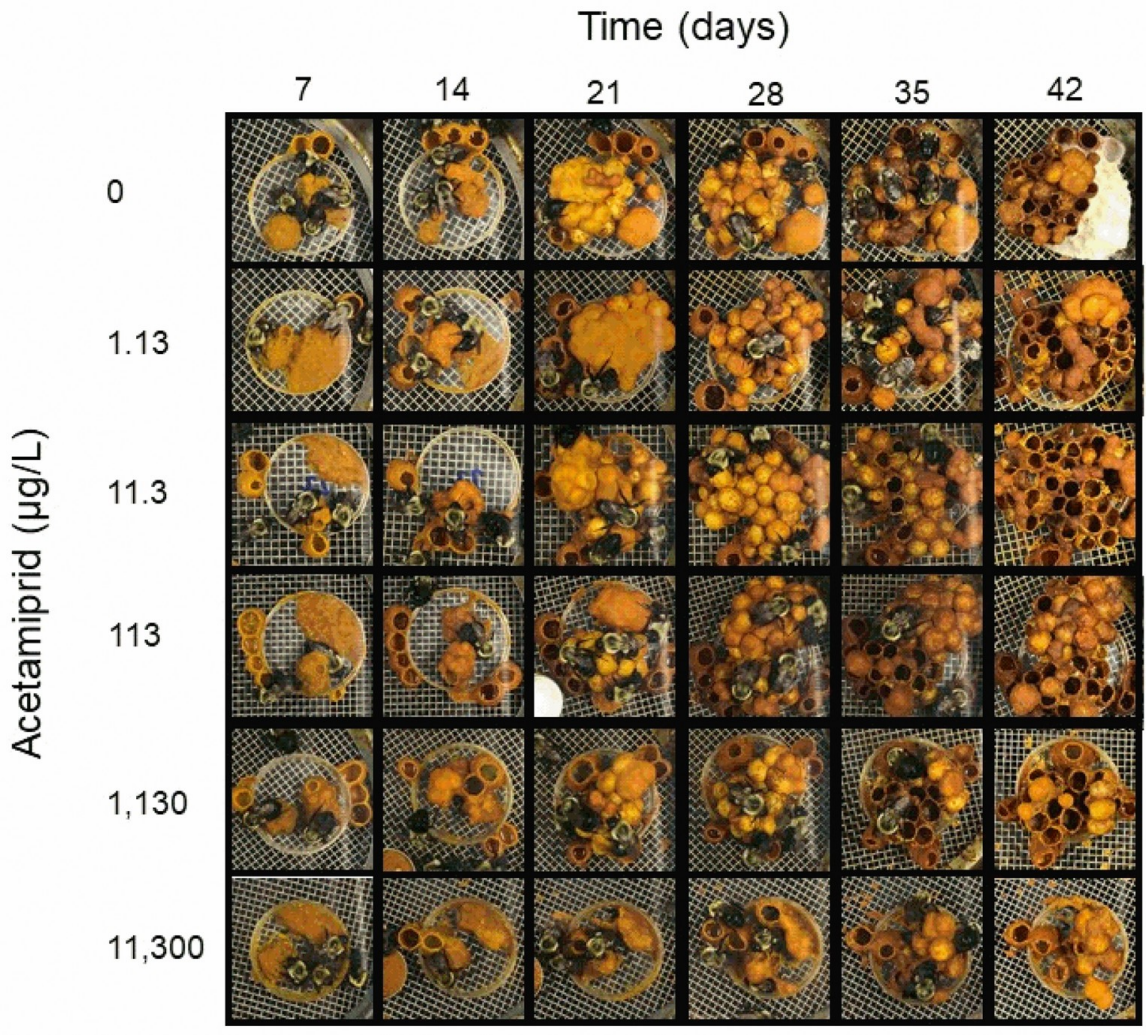
Concentration-related impacts of acetamiprid on microcolony growth and development. Representative photographs of microcolony development from week one (day 7) to experiment termination on day 42 in the presence of acetamiprid-containing sugar syrup. Representative photographs are shown from one of the ten microcolonies per treatment.

Microcolony development was observed daily in order to capture key developmental milestones, including the presence of an egg cup, capped egg chamber, brood masses, and pupal cells. In control microcolonies, nest developmental milestones were on average 3.0 ± 0.94 days for egg cup construction, 7.3 ± 0.95 days for capped egg chambers, 10.2 ± 1.9 days for brood masses, 21.3 ± 2.1 days for pupal cells. The highest concentration of acetamiprid (11,300 μg/L) significantly delayed egg cup construction (7 ± 1.2 days, p<0.05, One-Way ANOVA with Dunnett’s multiple comparisons test; Fig 2A), capped egg chambers (9.8 ± 1.4 days, p<0.05, One-Way ANOVA with Dunnett’s multiple comparisons test; Fig 2B), and time to pupal cells (31.25 ± 3.7 days, p<0.05, Kruskal-Wallis with Dunn’s multiple comparisons test, Fig 2D). Microcolony development was photographically documented weekly for one randomly selected microcolony for each group. Overall, nest size and complexity were impacted by acetamiprid exposure. Nests built in microcolonies exposed to the highest concentrations (1,130 and 11,300 μg/L) were noticeably smaller (Fig 3).

### Microcolony food consumption

**Fig 4:**
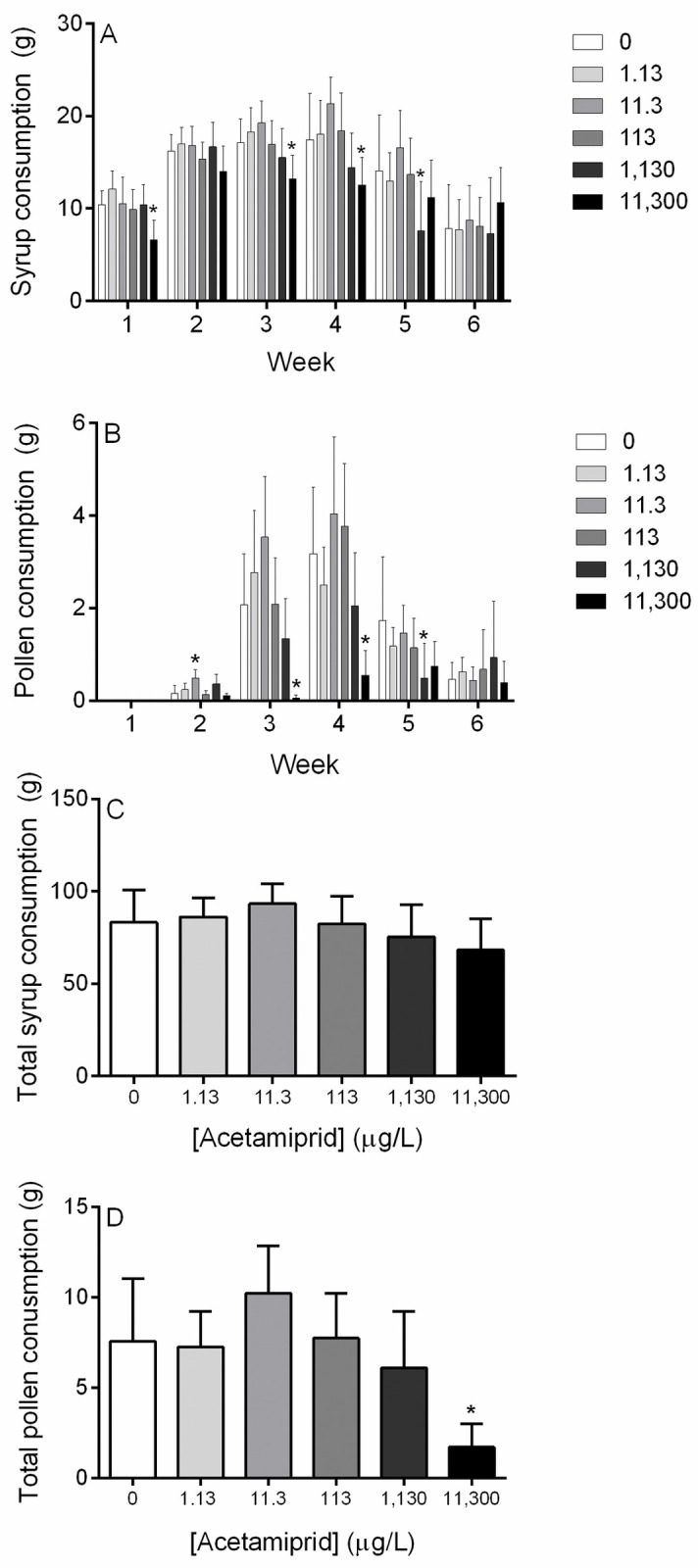
Microcolony syrup and pollen consumption. (**A**) Average syrup consumption by exposure group per week. (**B**) Average pollen consumption by exposure group per week. (**C**) Average syrup consumption over 6 weeks for each exposure group. (**D**) Average pollen consumption over 6 weeks for each exposure group. Pollen consumption tracking began during week two of the experiment. All values have been corrected for evaporation. Acetamiprid concentrations are expressed in μg/L. Data shown as mean ± SD (n = 10). * denotes p<0.05.

Overall, maximum syrup consumption occurred during week three and four for control microcolonies and for those exposed to lower concentrations of acetamiprid (1.13, 11.3, and 113 μg/L). The two highest concentrations (1,130 and 113,00 μg/L) followed a different trend, and their peak syrup consumption occurred in week two (Fig 4A). Syrup consumption was significantly decreased in the 11,300 μg/L group during week one, three, and four (p<0.05, One-Way ANOVA with Dunnett’s multiple comparisons test), and the 1,130 μg/L group during week five (p<0.05, One-Way ANOVA with Dunnett’s multiple comparisons test, Fig 4A).

Weekly pollen consumption, which was measured starting in week two of the experiment, also peaked during week three or four for all treatments except the highest concentration tested (11,300 μg/L, Fig 4B). Pollen consumption in the highest concentration group was significantly lower than control consumption during week three and four (p <0.05, Kruskal-Wallis with Dunn’s multiple comparisons test; Fig 4B).

Total syrup and pollen consumption over the course of the microcolony experiment were also calculated. No differences were found between total syrup consumption as compared to controls (Fig 4C). Total pollen consumption was significantly decreased in the highest exposure group (p<0.05, One-Way ANOVA with Dunnett’s multiple comparisons test; Fig 4D).

The amount of acetamiprid delivered to the microcolonies as a whole was estimated for each treatment (Table 1). The 11,300 μg/L microcolonies received on average 624.0 ± 153.0 μg of acetamiprid throughout the course of the 6 weeks. Consumption of acetamiprid by individual bees was also estimated, and individual workers in the 11,300 μg/L treatment group consumed on average 135.8 ± 25.95 μg of acetamiprid throughout the experiment (Table 1).

**Table 1:** Estimated acetamiprid utilized by whole microcolonies and individual workers.

Acetamiprid treatment (μg/L)	1.13	11.3	113	1,130	11,300
**Avg μg/MC**	0.078 ± 0.009	0.853 ± 0.098	8.73 ± 1.59	63.4 ± 15.4	624.0 ± 153.0
Avg μg/bee*	0.018 ± 0.001	0.191 ± 0.015	1.97 ± 0.35	16.4 ± 3.04	135.8 ± 25.95

*Adjusted for worker mortality.

### Drone production

**Fig 5:**
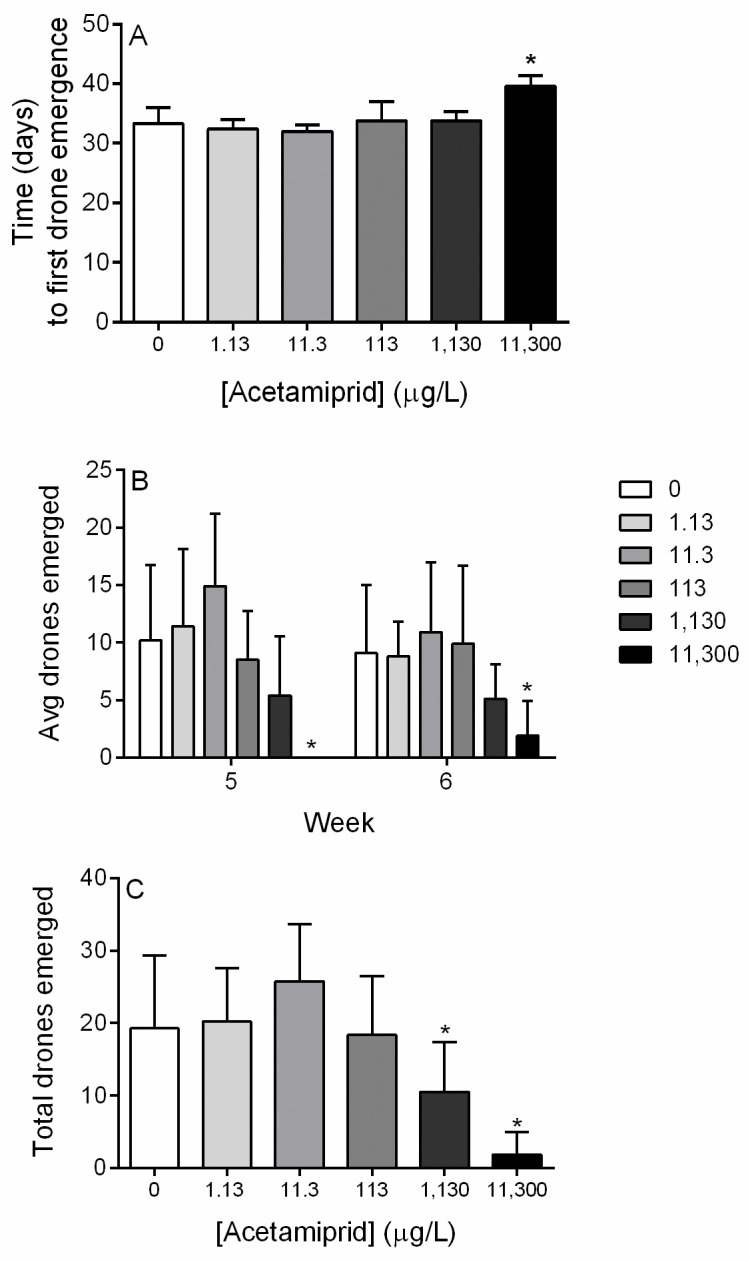
Microcolony drone production. Drones were collected from each microcolony as they emerged. (**A**) Time to first drone emergence by treatment group. (**B**) Average drone emergence in week five and six by treatment group. (**C**) Average drone emergence from microcolonies for the entire experiment. Acetamiprid concentrations are expressed in μg/L. Data shown as mean ± SD (n = 10). * denotes p < 0.05.

Control microcolonies produced their first drone on average 33.3 ± 2.7 days post-initiation. Drone emergence was significantly delayed for the highest concentration group (39.6 ± 1.8 days, p<0.05, Kruskal-Wallis with Dunn’s multiple comparisons test, Fig 5A). Drone emergence by week was examined and drone production was significantly decreased as compared to control for the highest concentration in both week five and six (p<0.05, Kruskal-Wallis with Dunn’s multiple comparisons test; Fig 5B). When total drone production was assessed, both 1,130 and 11,300 μg/L groups had significantly fewer drones as compared to controls (p<0.05, One-Way ANOVA with Dunnett’s multiple comparisons test; Fig 5C). Drones were weighed upon emergence, and no significant differences were found between average drone weight between groups (0 μg/L: 142 ± 28 mg, 1.13 μg/L: 144 ± 27, mg 11.3 μg/L: 136 ± 26 mg, 113 μg/L: 139 ± 28 mg, 1,130 μg/L: 144 ± 26 mg, 11,300 μg/L: 129 ± 42 mg; p>0.05, Kruskal-Wallis with Dunn’s multiple comparisons test).

### Analytical chemistry

Analytical chemistry of syrup samples via LC-MS had percent recovery ranging from 74 to 113.3% recovery (Table 2). The measured acetamiprid concentrations between the 0 and 48 hr samples for each concentration showed that degradation within the syrup was negligible, consistent with other analyses of acetamiprid in aqueous media [39] (cit). Therefore, measured concentrations were sufficiently similar to nominal concentrations.

**Table 2:** Analytical chemistry results of syrup containing acetamiprid.

Treatment group (μg/L)	Sample time	Measured (μg/L)	% as labeled
Control	0 hr	ND	NA
	48 hr	ND	NA
1.13	0 hr	1.3	112.4
	48 hr	0.8	74
11.3	0 hr	11.4	101
	48 hr	11.3	99.7
113	0 hr	128.1	113.3
	48 hr	126.1	111.5
1,130	0 hr	1,169.8	103.5
	48 hr	1,240.9	109.8
11,300	0 hr	10,971.0	97.1
	48 hr	10,679.6	94.5

## Discussion

Here we assessed the impact of acetamiprid exposure via syrup on *B*. *impatiens* microcolony growth and development. We found that the highest concentration assessed (11,300 μg/L) resulted in adverse effects on microcolony growth (delayed development), decreased pollen consumption, impaired nest building, and ultimately reduced nest productivity (drone production). We also observed alterations in worker behavior and worker mortality, however, the majority of mortalities occurred late in microcolony development and likely did not impact microcolony productivity outcomes. Lower acetamiprid exposure groups were unaffected at the concentrations tested, except for 1,130 μg/L, which also showed significantly reduced drone production. The no observable adverse effects level was determined to be 113 μg/L acetamiprid.

The pattern of mortality observed here was inverse of the effect seen on drone production. Control and low exposure concentration microcolonies experienced higher mortality but had no change in drone production, while microcolonies exposed to higher concentrations experienced lower mortality but had reduced drone production. Overall, the high incidence of mortality at control and lower concentrations did not appear deleterious to the microcolonies based on photographic documentation of nest development and nest productivity as measured by drone production, and was likely due to the random choice individual workers at the start of the experiment rather than an experiment effect. Further, the timing of mortalities, most of which occurred during the 4^th^ week of the experiment or later explain the lack of impact on nest productivity, as previous work with *B*. *impatiens* microcolonies has shown that capped egg chambers are not present in microcolonies after the second week of microcolony development [37], suggesting workers are no longer laying eggs. Further, at week 4 and beyond in microcolony development, reduced brood tending due to worker mortality would not impact offspring production, since most existing larvae would have transitioned to pupal cells by this time. Pupal cells do not require support from the workers beyond warmth, and since microcolonies were in a climate-controlled environment, this was likely not an important factor for pupation success. However, incubation temperature was below the temperature at which workers prefer to maintain brood, thus a developmental delay is possible [40]. One alternative explanation for this finding is that the mortality was related to pollen consumption, which was unaffected in control and lower concentrations and reduced at higher concentrations. It is possible that the corbicular pollen used here contained pollen from plants that decrease bee lifespan [41] or contained residues of other toxic chemicals, such as other insecticides and azole fungicides that act synergistically with neonicotinoids [30] and are routinely found in pollen matrices [32]. Detailed studies examining the effect of worker loss during a microcolony assessment has not been carefully studied and represents a current data gap.

We observed a significant delay in nest development milestones, including time to first egg cup formation, time to first capped egg chamber, as well as time to first pupal cell, in the microcolonies exposed to the highest acetamiprid concentration. Additionally, the high concentration nests were markedly smaller and less complex than control and low concentration nests. It is likely that both factors drove the observed reductions in drone production. Interestingly, the delays in development observed for the high exposure group were amplified over time. While average time to capped egg chambers was delayed by approximately 3 days in the high exposure group, delay in average time to first pupal cell formation was approximately 10 days. This may be indicative of slower larval growth or increased incidence of larval mortality within the high exposure group. However, it is important to note that the altered worker behavior observed at the highest concentration may have also contributed to delays in nest development and subsequent reduced drone production since workers are responsible for feeding the developing brood as well as building nest structures.

In evaluations of food consumption, there was no difference across treatment groups in total syrup consumed, regardless of concentration. This supports previous work that has shown that bumble bees do not find neonicotinoids within food sources unpalatable or aversive [28, 37, 42, 43]. Reductions in pollen consumption were observed at the high concentration (11,300 μg/L) and could be due to reduced foraging activity and reduced pollen consumption by workers as a result of the neurotoxic effects of acetamiprid, or due to reduced resourced requirements of the brood [27, 44]. Future studies using a foraging arena could help distinguish between effects of acetamiprid on adult behavior and resource requirements of the brood. Our observations of smaller nests resulting in diminished pollen consumption is consistent with previous microcolony assessments wherein toxicants that impact larval growth and development reduced pollen consumption [36, 37].

Within natural foraging environments, both bumble bees and honey bees consume plant nectar as their primary carbohydrate source. In the present study, acetamiprid was administered through sugar syrup to reflect a nectar exposure. Other neonicotinoids that are used as systemic pesticides, like imidacloprid and thiamethoxam, are transported to the nectar by plants at detectable levels [38, 45–48]. Acetamiprid, which is applied as a foliar spray rather than a seed coating, can be directly sprayed onto flowers and contaminate nectar and pollen [21, 31]. While that is the case, their residues are mainly found in pollen [32, 33, 49, 50]. Still, acetamiprid has been detected in nectar at concentrations ranging from 2.4 to 13 ppb in Poland [51], which is about 1400-fold lower than the high concentration tested here. While more assessments of acetamiprid in nectar are needed in other regions of the world, based on the available data, it is unlikely that environmentally relevant concentrations of acetamiprid in nectar will elicit the effects seen here.

With the microcolony design, establishing a per bee exposure is difficult since workers within the microcolony use syrup and pollen not only to feed themselves, but also to feed the developing brood and build nest structures [18]. Syrup is also stored by workers within honey pots, further obscuring the exact quantity of syrup consumed by the workers. Despite the challenges in determining a per bee dose, we estimated the amount of acetamiprid that individual workers encountered, since workers are a conduit through which all syrup utilized by the microcolony must move. We estimated that individual workers within the highest concentration group contacted on average 135.8 ± 25.95 μg of acetamiprid over the 6-week exposure period. Previous work conducted by our lab examined the impact of acetamiprid in pollen to *B*. *impatiens* microcolonies [37]. In that work, acetamiprid was mixed into pollen to create the pollen paste that was fed to microcolonies for a final concentration of 4250 μg/kg for the highest concentration tested. The similarity of the experimental designs allowed us to compare per bee exposures between the pollen and syrup routes of exposure. Comparisons revealed that the per bee acetamiprid exposure was about 18-fold higher with the syrup delivery. We also estimated the total amount of pesticide delivered to microcolonies as a whole, a practice with precedence in honey bee literature [52]. The highest treatment group here received on average 624.0 ± 153.0 μg acetamiprid. When compared to the pollen-based acetamiprid exposure we found that the syrup-based exposure yielded acetamiprid transfers to whole microcolonies that were about 9-fold higher [37]. Taken together, it suggests that in the wild, contaminated nectar may result in higher exposures to bumble bee nests than contaminated pollen.

Notably, the results of the pollen-based exposure and the present syrup-based exposure were similar wherein the highest concentration groups in both had impaired nest development and reduced drone production. However, behavioral impairments were observed at the two highest concentrations tested here, while the pollen-based acetamiprid exposures did not result in behavioral alterations [37]. The behavioral impairments in the present study may be due to differences in the amount of acetamiprid encountered by individual workers between the two studies as described above. As mentioned previously, adult workers can consume pollen, however, it is not a daily dietary requirement, whereas workers cannot survive for long periods of time without a carbohydrate source. With this is mind, it is likely that the workers in the present study consumed more contaminated diet than the pollen-based exposure since it was present as their primary source of carbohydrate. Conversely, it is likely that in the pollen-based exposure, while the microcolonies received less acetamiprid as a whole, a higher proportion of the pesticide was transferred to the brood, resulting in the reductions in drone production. This inference is supported by estimates of daily pollen and nectar requirements by adult workers and nonqueen larvae [53, 54]. Taken together these results highlight the important differences between these two routes of exposure and suggest that a pollen route of exposure may impact developing bumble bee brood more dramatically, while a nectar route of exposure may impact both workers and brood.

Our data suggest that at environmentally relevant concentrations, acetamiprid present in nectar (average 5.6 ppb (range 2.4–13 ppb) [51]) and pollen (up to 134 ppb [32, 33, 50]) would not result in adverse impacts to *B*. *impatiens* nest development. However, at high concentrations, acetamiprid has the potential to impede microcolony growth and development, as well as drone production, the indicator of nest productivity within the microcolony model. Additionally, we demonstrate the utility of the microcolony model for assessing developmental and population recruitment endpoints. These data, in conjunction with previously published data using a pollen exposure, provide foundational data on a common pesticide for a North American species of bumble bee. Whether, or not exposure to low levels of acetamiprid adversely impacts growth and development of other bumble bee species remains unknown.
